# Histone Demethylases JMJ30 and JMJ32 Modulate the Speed of Vernalization Through the Activation of *FLOWERING LOCUS C* in *Arabidopsis thaliana*

**DOI:** 10.3389/fpls.2022.837831

**Published:** 2022-07-01

**Authors:** Takashi Maruoka, Eng-Seng Gan, Nana Otsuka, Makoto Shirakawa, Toshiro Ito

**Affiliations:** ^1^Division of Biological Science, Graduate School of Science and Technology, Nara Institute of Science and Technology, Ikoma, Japan; ^2^Temasek Life Sciences Laboratory, National University of Singapore, Singapore, Singapore

**Keywords:** *Arabidopsis*, devernalization, epigenetics, *FLC*, histone demethylase, H3K27me3, JMJ30, vernalization

## Abstract

Vernalization is the promotion of flowering after prolonged exposure to cold. In *Arabidopsis thaliana*, vernalization induces epigenetic silencing of the floral repressor gene *FLOWERING LOCUS C* (*FLC*). Among the repressive epigenetic marks, the trimethylation of lysine 27 on histone H3 proteins (H3K27me3) is a critical contributor to the epigenetic silencing of *FLC*. The deposition of H3K27me3 is mediated by Polycomb Repressive Complex 2 (PRC2). Conversely, the elimination of H3K27me3 is mediated by histone demethylases, Jumonji-C domain-containing protein JMJ30 and its homolog JMJ32. However, the role of JMJ30 and JMJ32 in vernalization is largely unknown. In this study, we found that cold treatment dramatically reduced the expression levels of *JMJ30* and did not reduce those of JMJ32. Next, by using the genetic approach, we found that the flowering of *jmj30 jmj32* was accelerated under moderate vernalized conditions. Under moderate vernalized conditions, the silencing of *FLC* occurred more quickly in *jmj30 jmj32* than in the wild type. These results suggested that the histone demethylases JMJ30 and JMJ32 brake vernalization through the activation of *FLC*. Our study suggested that PRC2 and Jumonji histone demethylases act in an opposing manner to regulate flowering time via epigenetic modifications.

## Introduction

Flowering is a transition from vegetative growth to reproductive growth in the plant life cycle. Many annual plants flower after being exposed to warm conditions in spring following prolonged winter coldness (Chouard, 1960; Simpson and Dean, 2002). Acquisition of the ability to undergo flower-bud formation induced by the cold is referred to as vernalization. In a model plant, *Arabidopsis thaliana*, flowering is promoted by two pathways: (1) the vernalization pathway and (2) the autonomous pathway (Sheldon et al., 2000; Simpson and Dean, 2002; Michaels et al., 2005), and flowering is inhibited by the activity of a super transcriptional complex including the zinc finger protein FRIGIDA (FRI; Li et al., 2018). The vernalization pathway and autonomous pathway repress the expression levels of the floral repressor gene *FLOWERING LOCUS C* (FLC; Michaels and Amasino, 1999; Sheldon et al., 1999, 2000; Simpson and Dean, 2002), and they counteract *FRI*, which activates the expression of *FLC* during the development of plants (Johanson et al., 2000). *FLC* represses the two floral inducers *FLOWERING LOCUS T* (*FT*) and *SUPPRESSOR OF OVEREXPRESSION OF CONSTANS 1* (*SOC1*; Hepworth et al., 2002; Michaels et al., 2005; Helliwell et al., 2006; Searle et al., 2006). Therefore, stable silencing of *FLC* is essential during the induction of floral buds.

The vernalization pathway has a primary role in the regulation of *FLC*. First, cold treatment inactivates FRI by translocating from an active *FLC* locus to nuclear condensates, resulting in the downregulation of *FLC* (Whittaker and Dean, 2017; Zhu et al., 2021). Second, the vernalization pathway triggers a key repressive epigenetic modification of the *FLC* locus, including the trimethylation of lysine 27 of histone H3 (H3K27me3), in a stepwise fashion (Bastow et al., 2004; Whittaker and Dean, 2017). First, H3K27me3 is deposited at the nucleation region of *FLC* by Polycomb Repressive Complex 2 (PRC2) during vernalization. Second, after the transition from cold conditions to warm and long-day conditions, the repressive mark spreads to the entire *FLC* locus, and epigenetic marks are inherited after DNA replication/cell cycle progression. The spreading and maintenance of H3K27me3 on the *FLC* locus are dependent on LIKE HETEROCHROMATIN PROTEIN 1 (LHP1) and CURLY LEAF (CLF; Yang et al., 2017). In contrast to the deposition of H3K27me3 by PRC2, the elimination of H3K27me3 is regulated by histone demethylases. Five Jumonji-C-domain containing proteins (JMJs), JMJ11, JMJ12, JMJ13, JMJ30, and JMJ32, are reported to have H3K27me3 removal activity (Lu F. et al., 2011; Crevillen et al., 2014; Gan et al., 2014; Cui et al., 2016; Yan et al., 2018). Among them, we previously discovered that JMJ30 and JMJ32 are redundantly required for the prevention of extreme early flowering under high-temperature conditions (Gan et al., 2014). Under high-temperature conditions, JMJ30 and JMJ32 remove H3K27me3 from the *FLC* locus, resulting in upregulation of the expression levels of *FLC*. However, the roles of JMJ30 and JMJ32 in vernalization are still unclear.

Interestingly, we previously found that high temperature induced the expression of *JMJ30* and stabilized JMJ30 (Gan et al., 2014). In addition, the vernalized state can be canceled by short-term exposure to a high temperature. This is referred to as devernalization (Purvis and Gregory, 1945; Gregory and Purvis, 1948). In the model plant *Arabidopsis thaliana*, the expression levels of *FLC* are partially recovered after devernalization (Périlleux et al., 2013). H3K27me3 is erased at the *FLC* locus during devernalization (Bouché et al., 2015). We hypothesized that high temperature might erase H3K27me3 from the *FLC* locus via JMJ30. In this study, we tested this hypothesis by applying a genetic approach.

In this study, we examined whether JMJ30 and JMJ32 are involved in vernalization and devernalization in *Arabidopsis thaliana*. We found that JMJ30 and JMJ32 play a role in the molecular brake for vernalization and are not involved in devernalization. This study provides novel insights into the role of repressive histone marks in environmental responses in plants.

## Materials and Methods

### Plant Materials and Growth Conditions

All *Arabidopsis thaliana* seed stocks used in this study were in the *FRI*^*sf*–2^ (Lee et al., 1993) background except *pEstro:JMJ30* (Yamaguchi et al., 2021), which generated a Columbia (Col-0) background. *jmj30 jmj32* (Gan et al., 2014), *flc-3* (Michaels and Amasino, 1999), and the reporter line *FLC-GUS* (Noh and Amasino, 2003; Michaels et al., 2005) were reported previously. To generate multiple mutants and mutants harboring the reporters, we performed crossings and genotyping. *Arabidopsis* seeds were grown on 0.5% gellan gum or 1% agar with Murashige and Skoog (MS). The plates were cultivated under constant light conditions. To examine the flowering phenotypes, the plants were cultivated in pots containing vermiculite and Metro-Mix (Sun Gro Horticulture).

### Experimental Conditions for the Devernalization

We vernalized seeds 1 month after water absorption and sowed them on a plate. After vernalization, we transferred the plates to an incubator at 30° in the dark, cultivated them for 1 week, and then transferred them to a plate at 22°. For high reproducibility, incubation at 30° should be performed in the dark under our cultivation conditions.

### Reverse-Transcription Polymerase Chain Reaction and Quantitative Reverse-Transcription–Polymerase Chain Reaction

Samples were frozen in liquid nitrogen immediately. The RNeasy Plant Mini Kit (Qiagen, Hilden, Germany) was used to extract total RNA. The RNase-Free DNase Set (Qiagen, Germany) was used to eliminate the contamination of genomic DNA from the RNA samples. Reverse-transcription PCR was performed using PrimeScript RT Master Mix (Takara, Shiga, Japan). Quantitative RT–PCR was applied as described previously (Wang et al., 2020). Arabidopsis *PP2A* (for *FLC*) and *EIF4A* (for *JMJ30* and *JMJ32*) were used as the internal references. Each experiment was repeated at least three times. The relative expression level of each gene was calculated using the 2^–ΔΔCT^ method (Livak and Schmittgen, 2001). The primers are listed in Supplementary Table 1.

### β-glucuronidase Staining

Seedlings were fixed in 90% acetone for 30 min at room temperature and subsequently stained with β-glucuronidase (GUS) staining solution. The staining method was as described previously (Gan et al., 2014; Shirakawa et al., 2014). After GUS staining, samples were transparentized as described previously (Shirakawa et al., 2009). Representative images were photographed under an AXIO Zoom V16 (ZEISS) microscope.

### Flowering Phenotype Analysis

To test the timing of flowering, including the number of rosette or cauline leaves produced, we vernalized plants and then transferred them into soil cultivation conditions. We cultivated plants until the boltings of the primary stems and then counted the number of leaves, as described previously (Shirakawa et al., 2021).

### Chromatin Immunoprecipitation-Quantitative Polymerase Chain Reaction

Chromatin immunoprecipitation experiments were performed as previously described with minor modifications (Gan et al., 2014; Yamaguchi et al., 2014; Shirakawa et al., 2021). Briefly, total chromatin was extracted from the seedlings and immunoprecipitated using anti-H3K27me3 (Abcam, Cat. No. ab6002). The DNA fragments were recovered by QIAquick PCR Purification Kit (QIAGEN, Cat. No. 28106). qPCR with gene-specific primers (Supplementary Table 1) was performed on a LightCycler 480 System II (Roche) using a FastStart Essential DNA Green Master (Roche, Cat. No. 06924204001). Values of percent input of target loci were calibrated by values of percent input of *AGAMOUS* loci. The experiments were repeated three and six times for NV and V2W, respectively. The statistical significance was evaluated by two-tailed Student’s *t*-test.

### Data Statistics and Availability

In this study, one-way ANOVA followed by the Tukey–Kramer test or two-tailed Student’s *t*-test was performed to detect the differences as required.

## Results

### The Expression Levels of *JMJ30*, Not *JMJ32*, Were Gradually Reduced by Cold Treatment

**FIGURE 1:**
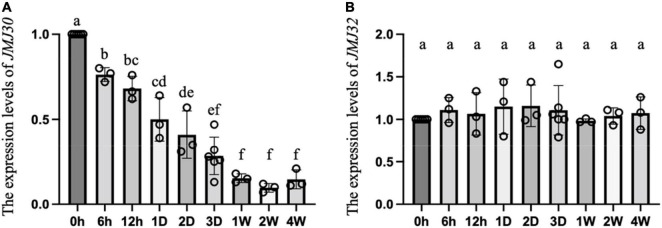
The gradual reduction in the expression levels of *JMJ30* during vernalization. The expression levels of endogenous *JMJ30* **(A)** and *JMJ32* **(B)** in seedlings at 3 days after germination. Seeds were treated with different lengths of cold (h, hour; D, day; W, week) in the dark after water absorption. Error bars represent SD. One-way ANOVA followed by the Tukey–Kramer test was performed (*p* < 0.05). Different letters indicate significant differences, while the same letters indicate non-significant differences.

First, we examined whether the expression levels of *JMJ30* and *JMJ32* were changed during vernalization. After water absorption by the seeds, we incubated them under various periods of cold treatment (from 0 h to 4 weeks) in the dark. Then, we germinated them on gellan gum plates and compared the expression levels of two genes, *JMJ30*, and *JMJ32*, in the seedlings at 3 days after germination (Figure 1). Interestingly, the expression levels of *JMJ30* started to decrease after 6 h of cold treatment, and they reached their minimum level after 1 week of cold treatment and were maintained at the minimum level for 4 weeks (Figure 1A; labeled “f” in one-way ANOVA followed by the Tukey–Kramer test). Unlike *JMJ30*, the expression levels of *JMJ32* were not changed by cold treatment (Figure 1B). These results suggested that a reduction of the expression levels of *JMJ30* occurred quickly after cold treatment; however, the activities of JMJ30 and 32 remained after cold treatments.

### *jmj30 jmj32* Exhibited an Early Flowering Phenotype Under Partial Vernalized Conditions

**FIGURE 2:**
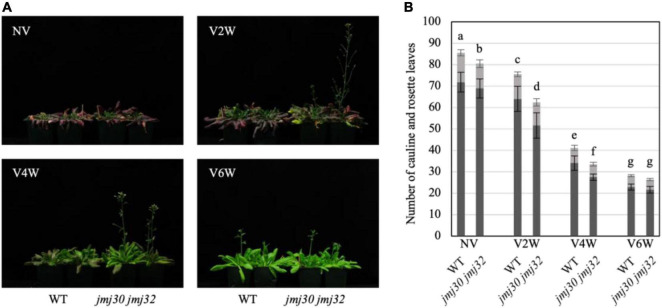
*jmj30 jmj32* exhibited the early flowering phenotype under the partial vernalized conditions. **(A)** Photographs of wild type and *jmj30 jmj32* under different vernalized conditions (NV, non-vernalization; V2/4/6W, vernalization at 2/4/6 weeks). **(B)** Number of leaves, including cauline (gray) and rosette (black) leaves. Error bars represent SD. One-way ANOVA followed by the Tukey–Kramer test was performed (*p* < 0.05). Different letters indicate significant differences, while the same letters indicate non-significant differences.

To clarify the roles of *JMJ30* and *JMJ32* in vernalization, we compared the flowering time between wild-type and *jmj30 jmj32* double mutants harboring the active *FRIGIDA* gene (hereafter, wild-type and *jmj30 jmj32*) (Figure 2). We did not test single mutants of *jmj30* and *jmj32* because they are redundantly required for the prevention of heat-induced extreme early flowering (Gan et al., 2014). In the non-vernalized conditions, *jmj30 jmj32* showed a slightly early flowering phenotype [Figure 2B; the total number of leaves: 85.45 (wild type) vs. 80.45 (*jmj30 jmj32*)]. Under the vernalized conditions of 2 weeks, *jmj30 jmj32* showed a clear early flowering phenotype [Figure 2B; the total number of leaves: 75.5 (wild type) vs. 62.35 (*jmj30 jmj32*)] because the difference in the total number of leaves was larger than that in the non-vernalized condition. Under the vernalized conditions of 4 weeks, *jmj30 jmj32* showed an early flowering phenotype [Figure 2B; the total number of leaves: 41.1 (wild type) vs. 33.5 (*jmj30 jmj32*)]; however, the difference in the total number of leaves was smaller than that under the vernalized conditions of 2 weeks. Finally, under the fully vernalized conditions of 6 weeks, *jmj30 jmj32* showed a similar timing of flowering as the wild type (Figure 2B). Collectively, these results suggested that JMJ30 and JMJ32 modulate the speed of vernalization.

### *jmj30 jmj32* Showed Reduced Expression Levels of *FLC* in the Partial Vernalized Conditions

**FIGURE 3:**
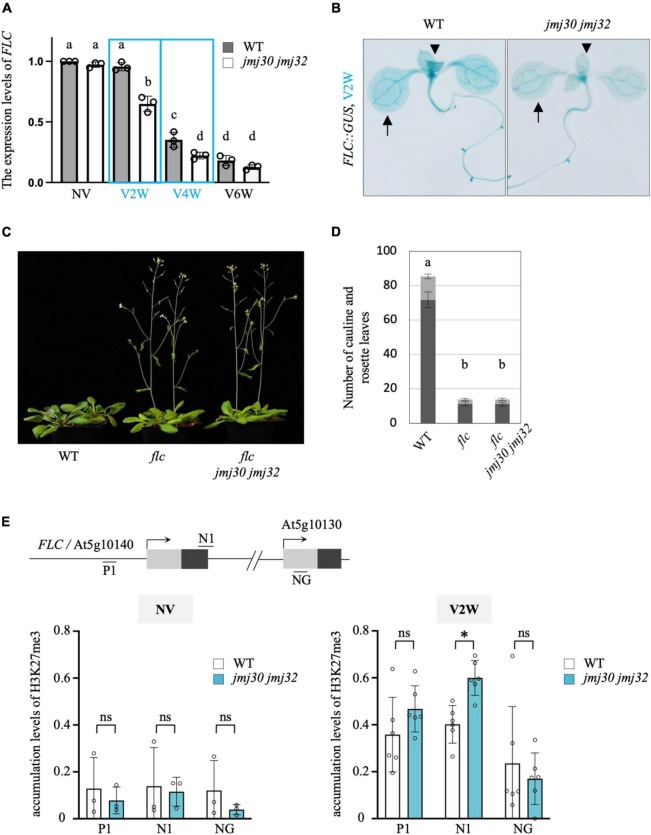
JMJ30 and JMJ32 modulate flowering time through the regulation of *FLC* during vernalization. **(A)** The expression levels of endogenous *FLC* of wild type and *jmj30 jmj32* in different vernalized conditions (NV, non-vernalization; V2/4/6W, vernalization at 2/4/6 weeks). Error bars represent SD. One-way ANOVA followed by the Tukey–Kramer test was performed (*p* < 0.05). Different letters indicate significant differences, while the same letters indicate non-significant differences. **(B)** GUS staining of seedlings of *FLC::GUS* and *FLC::GUS jmj30 jmj32* after vernalization for 2 weeks. Arrows indicate cotyledons, and arrowheads indicate true leaves. **(C)** Photographs of wild type, *flc*, and *flc jmj30 jmj32* in non-vernalized conditions. **(D)** Quantification of the flowering time of wild type, *flc*, and *flc jmj30 jmj32* under non-vernalized conditions. Number of leaves including cauline (gray) and rosette (black) leaves. Error bars represent SD. One-way ANOVA followed by the Tukey–Kramer test was performed (*p* < 0.05). Different letters indicate significant differences, while the same letters indicate non-significant differences. Note that *flc* and *flc jmj30 jmj32* showed an extreme early flowering phenotype to a similar extent. **(E)** Accumulation levels of H3K27me3 in wild type (white) and *jmj30 jmj32* (light blue) in the *FLC* locus under NV (left) and V2W (right) conditions. A schematic image of *FLC* locus and a neighboring gene (NG), At5g10130 was shown. Gray box indicates 5′-UTR and black box indicates first exon. N1 is located at the nucleation region of *FLC*. Note that in the V2W condition, higher levels of H3K27me3 were detected at N1 region in *jmj30 jmj32* than in the wild type. Values of percent input of target loci were calibrated by values of percent input of *AGAMOUS* loci. The experiments were repeated three and six times for NV and V2W, respectively. *p < 0.01; ns, not significant (two-tailed Student’s *t*-test).

Next, we examined whether JMJ30 and JMJ32 modulate the speed of vernalization through the expression levels of *FLC*. By quantitative polymerase chain reaction (qPCR) analysis, in the partially vernalized conditions (V2W and V4W), we found a significant reduction in *FLC* expression in *jmj30 jmj32* compared with the wild type (Figure 3A). In addition, we compared the spatiotemporal expression patterns of *FLC::GUS* between wild type and *jmj30 jmj32* (Figure 3B and Supplementary Figure 1). Under V2W and V4W conditions, the expression levels of *FLC* in both cotyledons and rosette leaves of *jmj30 jmj32* were lower than those in wild type. These results suggested that lower expression levels of *FLC* triggered the early flowering phenotype of *jmj30 jmj32* in the partially vernalized conditions. To clarify the genetic pathway between *JMJs* and *FLC*, we generated triple mutants, *flc jmj30 jmj32*. Under non-vernalized conditions, *flc* exhibited the extreme early flowering phenotype [Figures 3C,D; the total number of leaves: 85.45 (wild type) vs. 13.8 (*flc*)]. *flc jmj30 jmj32* also showed the extreme early flowering phenotype [Figures 3C,D; the total number of leaves: 13.85 (*flc jmj30 jmj32*)]. These results suggested that *FLC* is genetically epistatic to *JMJ30* and *JMJ32* in flowering. Combined with the data in Figures 3A,B, we concluded that *JMJ30* and *JMJ32* act upstream of *FLC*. Under high-temperature conditions, JMJ30 and JMJ32 are required for the elimination of H3K27me3 from the *FLC* locus. We examined whether the accumulation levels of H3K27me3 on the *FLC* locus were changed in *jmj30 jmj32* under partial vernalized conditions. We found that the accumulation levels of H3K27me3 on the nucleation region of the *FLC* locus were slightly but statistically significantly increased in *jmj30 jmj32* under partially vernalized conditions (V2W), while no clear changes were observed under non-vernalized conditions (NV) in multiple biological replicates (Figure 3E). Taken together, these results suggested that JMJ30 and JMJ32 modulate flowering time through the regulation of *FLC* during vernalization.

### Overexpression of *JMJ30* Can Confer the Late-Flowering Phenotype

**FIGURE 4:**
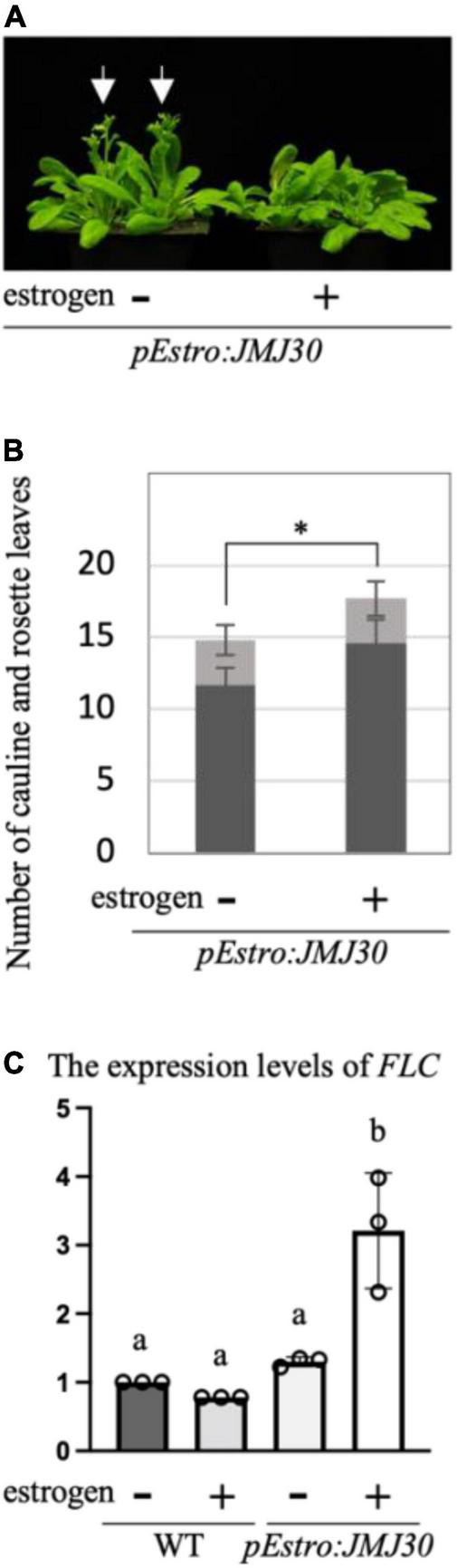
Overexpression of *JMJ30* conferred the late flowering phenotype. **(A)** Photograph of *pEstro:JMJ30* treated without (left) and with (right) estrogen. Arrows indicate the flowers. **(B)** Quantification of the number of cauline (gray) and rosette (black) leaves of *pEstro:JMJ30* treated without (left) and with (right) estrogen. Error bars represent SD. *p < 0.05 (Student’s *t*-test). **(C)** Quantification of the expression levels of *FLC* in wild-type and *pEstro:JMJ30* treated without (left) and with (right) estrogen. Error bars represent SD. One-way ANOVA followed by the Tukey–Kramer test was performed (*p* < 0.05). Different letters indicate significant differences, while the same letters indicate non-significant differences. Note that estrogen treatments did not alter the expression levels of *FLC* in the wild type; however, in *pEstro:JMJ30*, the same treatments upregulated the expression levels of *FLC*.

We generated transgenic plants, *pEstro:JMJ30* (Yamaguchi et al., 2021), in which *JMJ30* was overexpressed when we treated them with estrogen (Figure 4). In contrast to *jmj30 jmj32*, *pEstro:JMJ30* with estrogen treatment showed a slight late-flowering phenotype compared with the line without estrogen treatment (Figures 4A,B). Estrogen treatment induced upregulation of *FLC* (Figure 4C). These results suggest that overexpression of *JMJ30* may be able to confer the late-flowering phenotype through the regulation of *FLC*. *JMJ30* is one of the key factors regulating flowering time in Arabidopsis.

### The Devernalization Occurred in *jmj30 jmj32*

**FIGURE 5:**
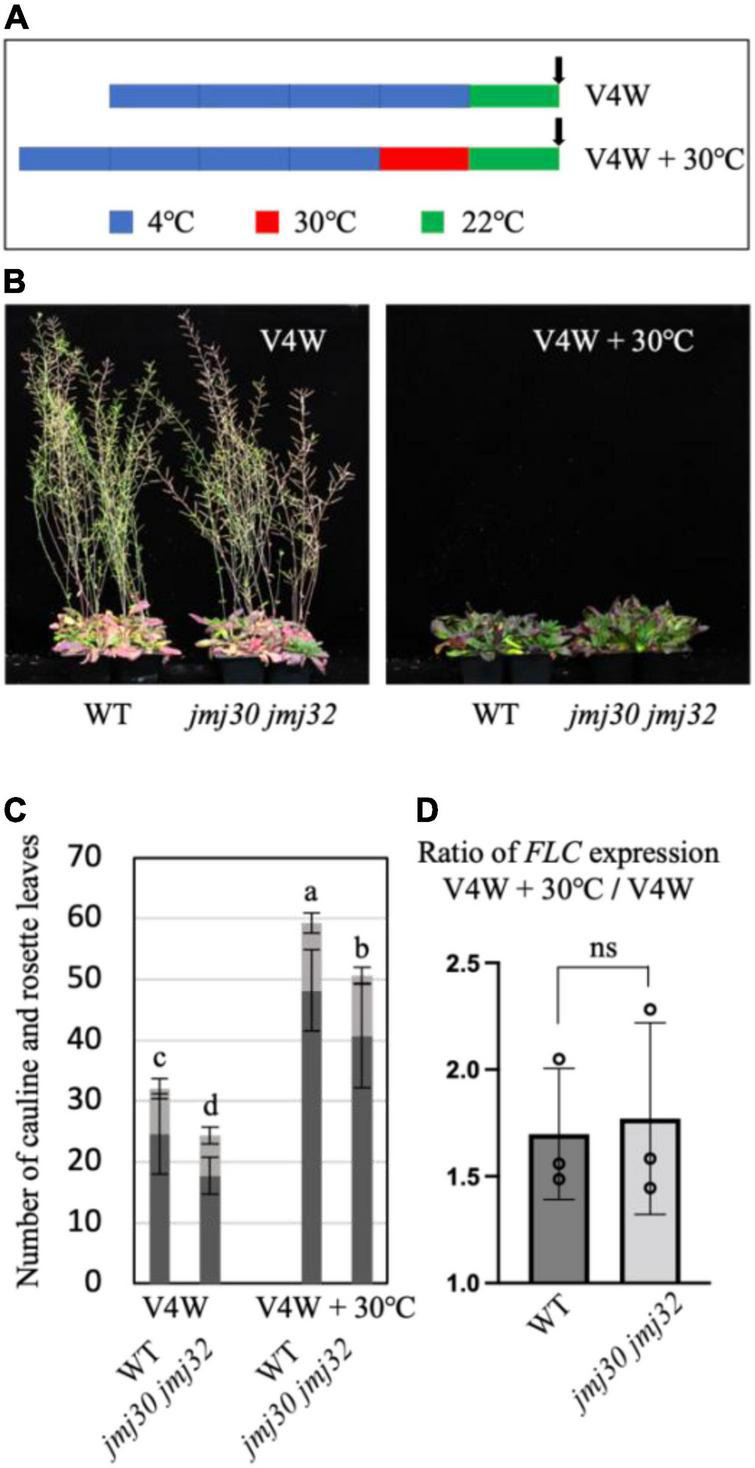
Devernalization occurred in *jmj30 jmj32.* **(A)** Schematic diagram of the experimental conditions. For the vernalized conditions (V4W), we incubated the seeds at 4° (blue) after water absorption and then cultivated them for a week at 22° (green) followed by sampling for RNA extraction. For devernalized conditions (V4W + 30°C), after vernalization, we transferred the plate at 30° in the dark (red) and then cultivated it at 22°. **(B)** Photographs of wild type and *jmj30 jmj32* in vernalized conditions (left) and devernalized conditions (right). **(C)** Quantification of the number of cauline (gray) and rosette (black) leaves of wild-type and *jmj30 jmj32* plants under vernalized conditions (left) and devernalized conditions (right). Error bars represent SD. One-way ANOVA followed by the Tukey–Kramer test was performed (*p* < 0.05). Different letters indicate significant differences, while the same letters indicate non-significant differences. **(D)** The ratio of *FLC* expression between V4W + 30°C and V4W. ns, not significant (Student’s *t*-test).

Devernalization is a reversion of vernalized status to non-vernalized status by heat. It was reported that H3K27me3 on the *FLC* locus was reduced after heat treatment (Bouché et al., 2015). In addition, we previously found that heat induced the upregulation of *JMJ30* and the stabilization of JMJ30 (Gan et al., 2014). Combining these results, we hypothesized that heat-activated JMJ30 might eliminate H3K27me3 from the *FLC* locus during devernalization. First, we established the experimental conditions for devernalization using Arabidopsis. We vernalized the seeds at 4° in the dark and then transferred them to 30° in the dark (Figure 5A). These plants showed a late flowering phenotype compared with vernalized plants [Figures 5B,C; the total number of leaves: 32.1 (V4W) vs. 59.3 (V4W + 30°C)]. Upon heat treatment, *jmj30 jmj32* showed a late flowering phenotype compared with vernalized *jmj30 jmj32* [Figures 5B,C; the total number of leaves: 24.3 (V4W) vs. 50.7 (V4W + 30°C)]. These results suggested that devernalization occurred even in *jmj30 jmj32*. Consistent with this, heat-treated *jmj30 jmj32* expressed 1.7-fold higher levels of *FLC* than vernalized *jmj30 jmj32*, as the wild-type did (Figure 5D). Taken together, these results suggested that *JMJ30* and *JMJ32* were not key factors for devernalization in Arabidopsis, although we could not exclude the possibility that JMJ30 and JMJ32 work with other histone demethylases during devernalization.

## Discussion

### JMJ30 and JMJ32 in the Vernalization Process

Histone demethylases involved in the vernalization pathway have not been identified. In this study, we showed that JMJ30 and JMJ32 act as molecular brakes for vernalization through the regulation of *FLC* in Arabidopsis (Figures 1–4). First, the loss-of-function mutants *jmj30 jmj32* exhibited an early flowering phenotype under partial vernalization conditions (Figure 2). Second, the levels of these early flowering phenotypes under different vernalized conditions were fairly consistent with the expression levels of *FLC*, and the genetic interaction with *FLC* indicated that JMJ30 and JMJ32 were upstream factors for *FLC* (Figure 3). Third, the deposition of H3K27me3 was enhanced in *jmj30 jmj32* at the *FLC* locus in partial vernalized conditions. Finally, the inducible overexpression of *JMJ30* caused the late-flowering phenotype via *FLC* regulation (Figure 4). Similar results were obtained by using a constitutive overexpression line of *JMJ30* (Gan et al., 2014). We found a cold-inducible reduction in *JMJ30* (Figure 1). To reduce the levels of *JMJ30*, plants may prepare for the start of vernalization. Future work will identify upstream factors for the cold inducibility of *JMJ30*.

### Molecular Mechanisms of Devernalization

In this study, we found that devernalization was triggered in *jmj30 jmj32* by heat, resulting in the upregulation of *FLC* and delayed flowering (Figure 5). These results suggested that *JMJ30* and *JMJ32* were not essential factors for devernalization in Arabidopsis, although we do not exclude the possibility that the other three jumonji proteins, JMJ11, JMJ12, and JMJ13, redundantly function in devernalization with JMJ30 and JMJ32 (Lu F. et al., 2011; Crevillen et al., 2014; Cui et al., 2016; Yan et al., 2018). It was reported that devernalized plants exhibited lower accumulation levels of H3K27me3 on the locus than vernalized plants (Bouché et al., 2015). However, it is largely unknown whether devernalization is induced by the active demethylation of *de novo* deposited H3K27me3. There are two additional targets of devernalization. For the stable silencing of *FLC* after vernalization, the spreading of H3K27me3 to the whole *FLC* genomic region and the maintenance of H3K27me3, *de novo* deposition of H3K27me3 into newly incorporated histones during cell division/DNA replication are required. Heat may inhibit these processes. LHP1 and CLF are required for the spreading and maintenance of H3K27me3 in the *FLC* locus and are not required for the deposition of H3K27me3 in the nucleation region of *FLC*, where H3K27me3 is deposited first after cold treatment (Yang et al., 2017). Interestingly, it was reported that *FLC* in *lhp1* and *clf* was slightly and gradually reactivated after vernalization. The levels of *FLC* reactivation are very similar to those in heat-induced (Périlleux et al., 2013) and chemical-induced reactivation (Shirakawa et al., 2021). It is interesting to question whether heat affects the activity and stability of LHP1 and CLF and whether devernalization responses occur in mutants. Future works involving time course analysis of H3K27me3 during devernalization will provide detailed insights into the molecular mechanisms of devernalization. Other epigenetic marks, such as H3K4me3 and H3K9me2 may be involved in devernalization.

### Multiple Roles of *JMJ30* and *JMJ32* in Arabidopsis

In their roles in flowering, *JMJ30* and *JMJ32* are required for abscisic acid (ABA) and brassinosteroid (BR) responses (Wu et al., 2019a,b, 2020), acclimation to high temperature (Yamaguchi, 2021a,b; Yamaguchi et al., 2021; Yamaguchi and Ito, 2021a,b), callus formation (Lee et al., 2018) and the regulation of period length (Lu S. X. et al., 2011). In addition, it has been reported that the expression of *JMJ30* and the stability of JMJ30 are regulated by heat (Gan et al., 2014). However, the upstream factors affecting JMJ30 expression and the stabilizer of JMJ30 are largely unknown. Future work will identify such factors. The functions of JMJ30 and JMJ32 in other plant species are still open questions to be addressed.

## Data Availability Statement

The original contributions presented in this study are included in the article/Supplementary Material, further inquiries can be directed to the corresponding authors.

## Author Contributions

TM, E-SG, MS, and TI conceived the study and revised the manuscript. TM, E-SG, and NO performed all the experiments. MS wrote a draft of the manuscript. All authors read and approved the final version of the manuscript.

## Conflict of Interest

The authors declare that the research was conducted in the absence of any commercial or financial relationships that could be construed as a potential conflict of interest.

## Publisher’s Note

All claims expressed in this article are solely those of the authors and do not necessarily represent those of their affiliated organizations, or those of the publisher, the editors and the reviewers. Any product that may be evaluated in this article, or claim that may be made by its manufacturer, is not guaranteed or endorsed by the publisher.
