# Supplementary material for: Histone Demethylases JMJ30 and JMJ32 Modulate the Speed of Vernalization Through the Activation of FLOWERING LOCUS C in Arabidopsis thaliana
Source: Front Plant Sci. 2022 Jul 1;13:837831. doi: 10.3389/fpls.2022.837831 (PMC9284024; doi:10.3389/fpls.2022.837831)
Supplement: Supplementary file 1 [file Presentation_1.pptx]

## Slide 1
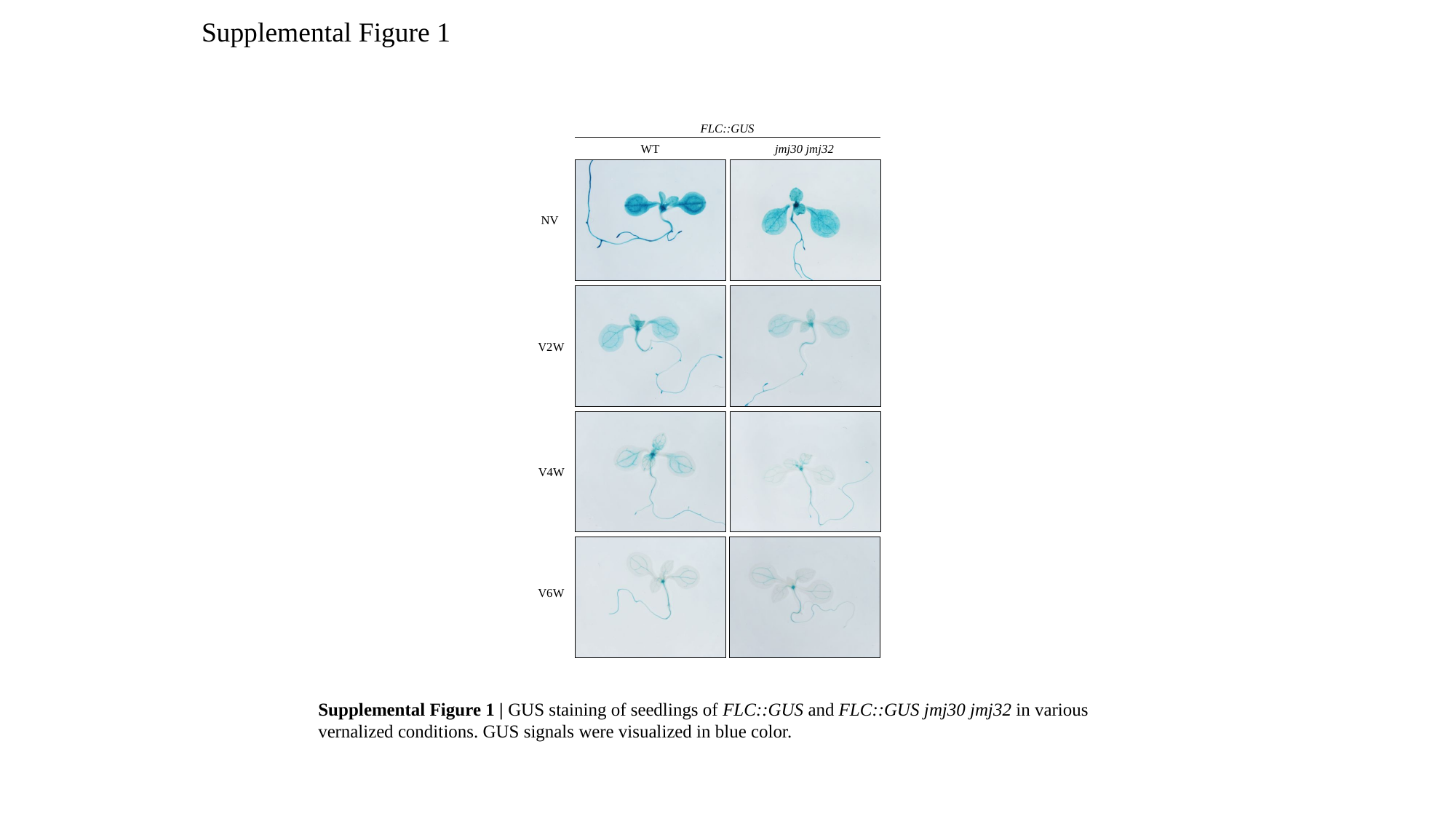

Supplemental Figure 1
FLC::GUS
WT
jmj30 jmj32
NV
V2W
V4W
V6W
Supplemental Figure 1 | GUS staining of seedlings of FLC::GUS and FLC::GUS jmj30 jmj32 in various vernalized conditions. GUS signals were visualized in blue color.
